# Using deep learning to screen OCTA images for hypertension to reduce the risk of serious complications

**DOI:** 10.3389/fcell.2025.1581785

**Published:** 2025-07-09

**Authors:** Yiheng Ding, Ziqiang Wei, Chaoyun Wang, Xinyue Li, Bingbing Li, Xueting Liu, Zhijie Fu, Hongwei Mo, Hong Zhang

**Affiliations:** ^1^ Eye Hospital, The First Affiliated Hospital of Harbin Medical University, Harbin, China; ^2^ School of Intelligent Science and Engineering, Harbin Engineering University, Harbin, China

**Keywords:** optical coherence tomography angiography, hypertension, deep learning, convolutional neural network, multi-Swin transformer

## Abstract

**Background:**

As a disease with high global incidence, hypertension is known to cause systemic vasculopathy. Ophthalmic vessels are the only vascular structures that can be directly observed *in vivo* in a non-invasive manner. We aim to investigate the changes in ocular microvessels in hypertension using deep learning on optical coherence tomography angiography (OCTA) images.

**Methods:**

The convolutional neural network architecture Xception and multi-Swin transformer were used to screen 422 OCTA images (252 from 136 hypertension subjects; 170 from 85 healthy subjects) for hypertension. Moreover, the separability of the OCTA images based on high-dimensional feature angles was analyzed to better understand how deep learning models distinguish such images with class activation mapping.

**Results:**

Under Xception, the overall average accuracy of 5-fold cross-validation was 76.05% and sensitivity was 85.52%. In contrast, the Swin transformer showed single-model (macular), single-model (optic disk), and multimodel average accuracies of 82.25%, 74.936%, and 85.06%, respectively, for predicting hypertension.

**Conclusion:**

The changes caused by hypertension on the fundus vessels can be observed more accurately and efficiently using OCTA image features through deep learning. These results are expected to assist with screening of hypertension and reducing the risk of its severe complications.

**Trial Registration:**

ChiCTR, ChiCTR2000041330. Registered 23 December 2020, https://www.chictr.org.cn/ChiCTR2000041330.

## 1 Introduction

Hypertension is an important public health problem that is responsible for the deaths of approximately 9.4 million people worldwide each year owing to complications like arteriosclerosis and stroke (Lim et al., 2012). According to the 2020 International Society of Hypertension Global Hypertension Practice Guidelines, hypertension is defined as systolic blood pressure equal to or greater than 140 mmHg and/or diastolic blood pressure equal to or greater than 90 mmHg (Unger et al., 2020). However, blood pressure measurements are often affected by many factors, including fluctuations at different time periods (Laski et al., 2000). Moreover, the simple blood pressure value cannot directly reflect the changes in systemic organs affected by hypertension. Hypertension can cause damage to the structure and functions of blood vessels, especially those like the microvessels in the eye (fundus retinal vessels) and glomerular capillaries (Laski et al., 2000). In the clinic, compared to simple blood pressure measurements, changes in the blood vessels caused by hypertension can better reflect the impacts on important organs and indicate the risk of serious complications of hypertension. Therefore, observation of systemic vascular changes is significant for early detection and treatment of hypertension and enhances the medical compliance of patients. Retinal vessels can be directly observed *in vivo* in a non-invasive manner and reflect changes in the circulatory system.

Hypertension can cause a series of pathophysiological changes in the eye, and the most common among these is hypertensive retinopathy (Keith et al., 1974). Hypertensive retinopathy is caused by acute and/or chronically elevated blood pressure and reflects the severity as well as duration of such elevated blood pressure status (Ambresin and Borruat, 2015; Stryjewski et al., 2016; Ahn et al., 2014; Tso and Jampol, 1982), which in turn indicate increased risks associated with stroke (Ong et al., 2013), congestive heart failure, and cardiovascular death (Tso and Jampol, 1982). Ong et al. (2013) found that persons with moderate hypertensive retinopathy were more likely to experience stroke than those with no retinopathy (multivariable hazard ratio (HR) for moderate vs. no retinopathy: 2.37, 95% confidence interval (CI): 1.39–4.02) after adjusting for age, sex, blood pressure, and other risk factors. In hypertensive subjects who had achieved good control of blood pressure with medication, hypertensive retinopathy was related to increased risk of cerebral infarction (HR for mild retinopathy: 1.96, 95% CI: 1.09–3.55; HR for moderate retinopathy: 2.98, 95% CI: 1.01–8.83) (Ong et al., 2013). This suggests that hypertensive retinopathy could predict the long-term risk of stroke independent of the blood pressure even in treated hypertensives with good control (Ong et al., 2013). At present, the main approaches for observing hypertensive retinopathy are via ophthalmoscope and fundus photography. Generally, only the central retinal vessels (grade 1 and 2 branches of the central retinal artery and vein) can be observed by these methods. However, early angiopathy caused by hypertension often manifests at the microvasculature level (grade 3 and 4 branches of the central retinal artery and vein) (Wong and Mitchell, 2004). Thus, obvious vascular changes are often not observable in patients in the early stages of hypertension (Wong and Mitchell, 2004). Moreover, most of the available research results are based on the judgments of observers, which are not objective enough (Wong and Mitchell, 2004). A previous work reported the development of a new computer-based program with innovative fundus photography and computational image processing technology to quantitatively evaluate the retinal geometry and branching parameters, such as curvature, fractal dimension, branch angle, and vascular aspect ratio (Cheung et al., 2012). However, the accuracy of this approach was based on manual identification, and only larger blood vessels could be observed via fundus photography. Moreover, the abovementioned geometric branch parameters are relatively independent low-dimensional features.

Optical coherence tomography angiography (OCTA) is a rapid and non-invasive diagnostic imaging technique that can produce depth stratified and high-resolution images of the retinal microvascular system without dye injection, thereby avoiding problems such as allergies caused by fundus fluorescein angiography. This allows observation of relatively smaller vascular structures and quantitative analyses of the findings (Spaide et al., 2015). Using built-in automatic OCTA software, we can easily evaluate retinal blood flow based on various retinal and choroidal microvascular parameters, such as vessel density, choroidal capillary flow area, foveal avascular zone (FAZ) area, and capillary density of the optic nerve head (ONH). Recently, several OCTA studies have shown that hypertension causes changes in the retinal microvascular system. An earlier study showed that changes in the foveal blood flow density were closely related to the Keith–Wagener–Barker grade (Takayama et al., 2018). In hypertensive patients without hypertensive retinopathy, the blood flow density of the superficial macular area was lower, and the retinal nerve fiber layer (RNFL) was thinner than those in the healthy control group (Hua et al., 2020). Another study showed that the RNFL was thinner in hypertensive patients than in healthy controls as the retinal blood flow density and perfusion density were lower in hypertensive patients (Lim et al., 2019). Moreover, the blood flow densities in patients with severe hypertensive retinopathy were lower than those of healthy controls (Lee et al., 2019).

Deep learning has been applied in the field of ophthalmology to screen and diagnose cataracts (Gao et al., 2015), keratopathy (Smadja et al., 2013), and retinal diseases (Ting et al., 2017). Traditionally, convolutional neural networks (CNNs) have been used as deep-learning architectures to achieve great results in all areas of computer vision. However, given the limitations of the convolutional structure, CNNs still have disadvantages in terms of the locality of the perception field even with increased network depth. This prevents the CNN from being able to obtain long-distance information between key feature points when extracting image features. Recently, transformer networks have been shown to generally perform better than CNNs in computer vision applications. One reason for this is that the self-attention mechanism in the transformer network can extract long-distance features and key information from global data. Just as recurrent neural networks have solved the problem of long-distance forgetting in text recognition tasks, transformer networks have provided similar advantages with respect to images.

Transformer networks require greater amounts of data than CNNs for training the random initialization network to achieve better results owing to the complexity of the network structure. However, acquiring high-quality labeled data is often very expensive in the field of medical research. Therefore, medical image datasets having the same sizes as natural image datasets are difficult to collect, which motivates the question whether transformer networks are unsuitable for clinical medical research. A recent study by Christos et al. (2021) showed that it is possible to obtain results similar to those with CNNs for medical problems by importing ImageNet pretraining weights into the transformer network through transfer learning; the study also demonstrated the feasibility of transformer networks for small-sample problems in the medical field.

For problem of With regard to OCTA images and hypertension, previous studies have shown that hypertension can affect the density distribution of blood vessels in the patients’ eyes. Considering the specificity of this problem, we need a deep-learning model that can capture key long-distance information from global blood vessel features and associated data, for which transformer networks may be better choices. In the subsequent sections, we compare some classical CNNs with the transformer network.

## 2 Methods

### 2.1 Data preparation

#### 2.1.1 Study participants

We recruited 136 patients (252 eyes) with hypertension diagnosed by a physician and 85 healthy volunteers (170 eyes) for the present study (Table 1). The hypertension subjects met the diagnostic criteria of essential hypertension with no history of other systemic diseases (such as hyperglycemia, stroke, hematological diseases, and autoimmune diseases). Furthermore, the healthy subjects in the control group had no reported history of systemic diseases. The subjects in both groups had no history of other intraocular diseases, such as diabetic retinopathy, macular edema, macular hole, epimacular membrane, age-related maculopathy, retinal detachment, retinal vascular occlusion, central serous chorioretinopathy, uveitis, and optic nerve disease; moreover, there was no history of intraocular surgery, intraocular injection, fundus laser, and ocular trauma. They had no glaucoma or suspected glaucoma (large cup–disc ratio, asymmetric cup–disc ratio, defective or narrow rim, optic disc hemorrhage, or suspected changes in the nerve fiber layer) and were not first-degree family members of non-glaucoma patients. We performed comprehensive physical and eye examinations for the patients, including systemic examination, best-corrected visual acuity (BCVA), intraocular pressure (IOP), computer optometry, slit lamp examination, fundus photography, and OCTA. The exclusion criteria included the following:

(1) primary or other secondary retinal diseases;
(2) inability to complete OCTA examination;
(3) severe corneal leukoplakia, cataract, strabismus, nystagmus, etc.;
(4) severe ametropia (>+ 300 or <−600);
(5) IOP not within the range of 10–21 mmHg;
(6) OCTA scan quality <7/10.

**TABLE 1:** Datasets of the patient and eye parameter distributions.

Patient		Normal (n = 85)	Hypertension (n = 136)	*p* value*
	Age (SD)	52.82 (11.35)	52.08 (10.715)	0.703
	Male/Female	37/48	68/68	0.349
Eye		Normal (n = 170)	Hypertension (n = 252)	*p* value*
	IOP (SD)	15.701 (2.2299)	15.909 (2.3725)	0.159
	BCVA, logMAR (SD)	0.04235 (0.04941)	0.05159 (0.04997)	0.063

The Fisher exact test was used for the classified variables (gender), while the Wilcoxon rank-sum test was used for continuous variables (age); IOP: intraocular pressure; BCVA: best-corrected visual acuity; the standard t-test was used for IOP and BCVA, and the difference was deemed statistically significant at *p* < 0.05.

This study was approved by the ethics committee of the First Affiliated Hospital of Harbin Medical University, Harbin, China (approval no. 2020151) and was registered with the China Clinical Trial Center (no. ChiCTR2000041330). All procedures were conducted in accordance with the guidelines of the Declaration of Helsinki.

#### 2.1.2 Image acquisition

The RTVue imaging device (Optovue, Inc., Fremont, CA, United States) was used to scan the macular area with 6 × 6 mm angiography and optic disc with 4.5 × 4.5 mm angiography in all eyes without mydriasis to obtain images of the microvascular system in the superficial layer of the macular retina and retinal posterior capillary. The instrument was operated at a central wavelength of 840 nm and speed of 68,000 scans per second, where each B scan involved 245 scans in both the horizontal and vertical directions. The microangiography composite algorithm was used to analyze complex signal changes (intensity and phase changes are included in the continuous B scan at the same position) and generate the microvascular image. We used the AngioVue® OCTA software to analyze all scans.

### 2.2 Data preprocessing

**FIGURE 1:**
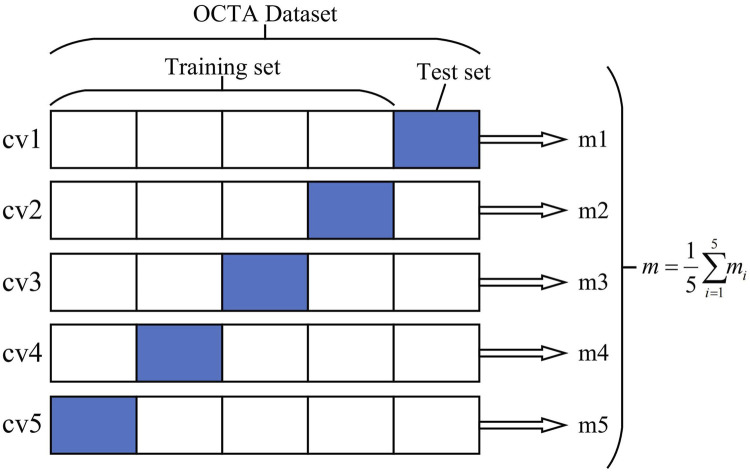
Optical coherence tomography angiography (OCTA) image dataset cross-validation diagram. The OCTA dataset is divided into five independent parts, of which four are taken as the training set and one part is used as the test set each time. Finally, the average index of the five models is taken as the overall model evaluation index.

The dataset was first divided into training and test sets in the ratio of 8:2. The sample categories in the training set, test set, and total dataset were the same. To minimize the evaluation differences caused by the size of the dataset, we mainly adopted two measures:

1) Perform data augmentation on the OCTA images in the divided training set, such as random flip, rotation, scaling, and noise processing. Each image in the training set of the OCTA image dataset was augmented by a factor of 10, such that the total size of the training set was 3,380 OCTA images.
2) Five-fold cross-validation (cv) was used to obtain five cross-training tests on each dataset, and the final average evaluation index was taken as the final index to minimize the evaluation differences between samples. Figure 1 schematically illustrates the division of the dataset into training and test samples.

### 2.3 Neural network architecture

The OCTA dataset used in this study contained 422 images, which resulted in the small-sample problem. To mitigate the overfitting problem that may occur during neural network training, we used transfer learning to transfer the model weights of large natural datasets to the target model to improve model robustness. After transferring the weights, we input the training images of the OCTA dataset to the target model for fine-tuning.

We then selected the Swin transformer as the baseline model to facilitate improvement in subsequent experiments (Liu et al., 2021) and perform comparisons with some classical CNN models; we also compared the effectiveness of each algorithm through 5-fold cross-validation and analysis of the evaluation indicators. The model training involved migration learning, where the pretraining weights were imported from ImageNet into the target model for fine-tuning the downstream tasks. No additional processing steps were used to compare the effectiveness of the algorithms.

After selecting the baseline model, we used multimodel fusion and prior knowledge initialization to improve the model. Then, visual analysis was performed to observe the locations of lesions caused by hypertension in the OCTA images, and the lesion areas were analyzed based on prior medical knowledge.

**FIGURE 2:**
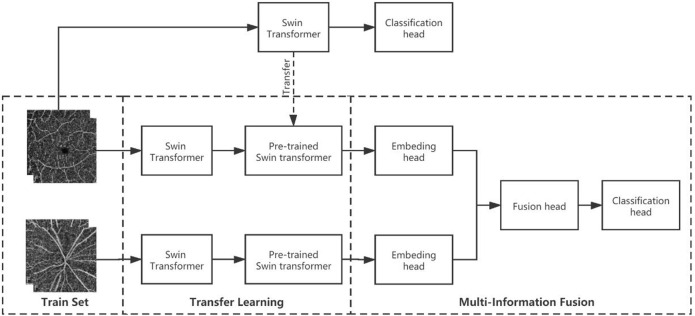
Multimodel-fusion-based information fusion of data from the macular and optic disc areas of the OCTA image. During training, the macular and optic disc images are imported into two separate Swin transformer branches. Then, the macular model branch adopts the pretraining weights of the macular arm (trained on only macular images), while the visual panel model branch adopts the pretraining weights from ImageNet (pretrained on large natural images). Finally, the embedding heads of the two model branches are connected in series as the information fusion head for training.

The multimodel fusion network used here is called multi-Swin as it is composed of two branches for Swin-transformer-based late fusion. In the diagnosis of hypertension, the macular area is mainly used to observe changes in the micro blood vessels and FAZ area, while the optic disc region is used to observe changes to the relatively larger vessels; the cup–disc ratio can also be used to judge the damage to the nerves. Hence, we combined observations from these two areas to make a more comprehensive judgment. The model structure has two branches, so the images from the macular and optic disc areas are input into these two branches separately. Considering that the image information in the two branches is not the same, we adopted a weight training strategy without sharing to fully extract the data from the two branches. Figure 2 shows the structure of the model used in this study.

We made some changes to the multimodel fusion initialization because the multi-Swin model predicts hypertension by integrating directly obtained image information from the macular and optic disc areas.

First, we input the OCTA images of the macular and optic disk areas to the Swin transformer networks separately for training and obtaining the “prior knowledge” weights; then, we attempted four different initialization methods for multimodel fusion, namely double ImageNet pretraining, macular pretraining + ImageNet pretraining, optic disc pretraining + ImageNet pretraining, macular pretraining + optic disc pretraining. Through these approaches, we expect that one of the branches can obtain prior knowledge before multimodel fusion to help the other branch extract features better.

### 2.4 Evaluation metrics

In our experiments, the following indices were used to evaluate the diagnostic performance of the model: accuracy (Acc), sensitivity (Se), specificity (Sp), precision (Pr), recall, and F1-score (F1). The calculation formulas for these metrics are as follows:

Accuracy: $Acc=\left(TP+TN\right)/\left(TP+FP+TN+FN\right)$ ,
Sensitivity: $Se=TP/\left(TP+FN\right)$ ,
Specificity: $Sp=TN/\left(FP+TN\right)$ ,
Precision: $\mathrm{Pr}=TP/\left(TP+FP\right)$ ,
Recall: Re=Se , and
F1-score: $F1=2*\mathrm{Pr}*\text{Re}/\left(\mathrm{Pr}+\text{Re}\right)$ ,

where true positive (TP) denotes the model that correctly predicts the OCTA image of a patient with hypertension; true negative (TN) represents the model that correctly predicts the OCTA image of a normal person; false positive (FP) refers to the model that mistakenly predicts the OCTA image of a normal person as a hypertensive patient; false negative (FN) refers to the model that mistakenly predicts the OCTA image of a hypertensive patient as a normal person. Furthermore, the receiver operator characteristic curve and area under the curve (AUC) were adopted to evaluate the network classification ability.

### 2.5 Equipment and statistical analysis

The experimental deep neural network was established on Keras’s deep-learning framework; the machine learning tool Scikit-Learn based on Python was used to interact with Keras’s interface. The operating system used was Ubuntu16.04, and the hardware platform was configured with an Intel Xeon (R) CPU E5-2620v3@2.4GHzx12 processor and Nvidia GeForce GTX1080Ti graphics card.

The quantitative variables were described in terms of the mean ± standard error of the mean, and the Shapiro–Wilk test was used to assess data normality. For comparison between groups, the standard t-test was used when the quantitative variables obeyed the assumption of homogeneity of variance of the normal distribution and the Wilcoxon rank-sum test was used otherwise. The qualitative variables were described via frequency and percentage, and the Fisher exact test was used for comparisons between groups. The differences were considered to be statistically significant at *p* < 0.05.

## 3 Results

### 3.1 Baseline model

To verify our previous judgment, we compared the training results of the Swin transformer network and classical CNNs for the macular OCTA images. Based on five-fold cross-validation, the accuracy evaluation results are as shown in Table 2.

**TABLE 2:** Comparison of accuracy evaluations between the transformer and classical convolutional neural network.

K fold	Swin-T	Xception (Chollet, 2017)	EfficientNet-B3 (Tan and Le, 2019)	ResNet50 (He et al., 2016)
F1	**82.50%**	75.41%	70.00%	66.25%
F2	**83.75%**	73.93%	77.50%	71.25%
F3	**75.00%**	72.12%	71.50%	67.50%
F4	**86.25%**	79.40%	72.25%	71.25%
F5	**83.75%**	79.40%	75.00%	70.00%
Average	**82.25%**	76.05%	73.25%	69.25%

Comparisons of the accuracies between the transformer and classical convolutional neural networks through 5-fold cross-validation. The values shown in boldface in the table represent the maximum values of those rows.

It is seen from this table that for the divided five-fold dataset, the transformer model achieves greater accuracy. To further verify the effectiveness of the transformer network for identifying hypertension, we choose the Xception and transformer models with better effects with the classical CNN architecture to compare other evaluation indices, whose results are shown in Table 3.

**TABLE 3:** Comparison of other evaluation indices between the Swin transformer and Xception models.

K fold	Swin transformer					Xception				
	ACC	SE	SP	F1-score	AUC	ACC	SE	SP	F1-score	AUC
F1	82.50%	89.58%	71.88%	0.8600	0.8710	75.41%	84.51%	61.76%	0.8049	0.8047
F2	83.75%	85.42%	81.25%	0.8632	0.8711	73.93%	87.80%	67.06%	0.8354	0.7906
F3	75.00%	85.42%	59.38%	0.8039	0.7454	72.12%	86.08%	51.18%	0.7874	0.7906
F4	86.25%	89.58%	81.25%	0.8866	0.9329	79.40%	83.60%	59.71%	0.7924	0.7931
F5	83.75%	83.33%	84.38%	0.8602	0.8548	79.40%	85.60%	70.29%	0.8319	0.8607
Average	**82.25%**	**86.67%**	**75.63%**	**0.8548**	**0.8550**	76.05%	85.52%	62.00%	0.8104	0.8249

ACC: accuracy; Se: sensitivity; Sp: specificity; F1: F1-score; AUC: area under the curve. The values shown in boldface in the table indicate the maximum values for the indices.

It is seen from Table 3 that the five-fold cross-validation of the Swin transformer model is better than the Xception model for all evaluation indices, which fully validates the effectiveness of the transformer model.

### 3.2 Multi-Swin transformer

We first trained the images from the macular and optic disc areas separately to obtain the prior knowledge weights required to initialize the multimodel fusion network. The model accuracies of the two sets of images are shown in Table 4.

**TABLE 4:** Comparison of accuracy evaluation indices between the macular and optic disk models.

K fold	Single model (macular)	Single model (optic disk)
F1	**82.5%**	75.90%
F2	**83.75%**	71.08%
F3	**75%**	77.11%
F4	**86.25%**	83.13%
F5	**83.75%**	75.90%
Average	**82.25%**	76.62%

Comparisons of the accuracies of the macular and optic disk models through 5-fold cross-validation. The values shown in boldface in the table represent the maximum values of the rows.

After obtaining the prior knowledge weights, we designed four comparative experiments to analyze the effects of multimodel fusion under different prior knowledge weights, as shown in Table 5.

**TABLE 5:** Comparison of the four multimodel experiments.

K fold	Double ImageNet	Macular + ImageNet	Optic disk + ImageNet	Macular + optic disk
F1	81.93	**83.13**	80.72	80.72
F2	81.93	**83.13**	80.72	81.93
F3	77.11	**78.31**	75.9	77.11
F4	86.75	**91.57**	84.34	89.16
F5	85.54	85.54	**89.16**	86.75
Average	82.652	**84.336**	82.168	83.13

ImageNet represents the ImageNet pretraining weights, Macular represents the “prior knowledge” weights obtained from pretraining with macular images, and Optic Disk represents the “prior knowledge” weights obtained from pretraining with optic disc images. The values shown in boldface in the table represent the maximum values of the rows.

From this table, it can be concluded that the multi-Swin model is improved to various degrees by the addition of different prior knowledge weights, which makes the model closer to the global optimal point and prevents it from being trapped in the local optimal point while speeding up model convergence.

### 3.3 Multi-Swin result analysis

From the above experiments, we obtain five model weights that are optimal for the five-fold cross-validation. To verify the effectiveness of the multimodel method, we compared the multi-Swin network with the Swin transformer network for various evaluation indices, and the results are shown in Table 6.

**TABLE 6:** Comparison of the single-model (macular) and multimodel training indices.

K fold	Swin transformer					Multi-Swin				
	ACC	SE	SP	F1-score	AUC	ACC	SE	SP	F1-score	AUC
F1	82.50%	89.58%	71.88%	0.86	0.8710	83.13%	88%	75.76%	0.78	0.8624
F2	83.75%	85.42%	81.25%	0.86	0.8711	83.13%	88%	75.76%	0.78	0.84
F3	75.00%	85.42%	59.38%	0.80	0.7454	78.31%	84%	70.11%	0.72	0.7745
F4	86.25%	89.58%	81.25%	0.89	0.9329	91.57%	92%	90.9%	0.89	0.9552
F5	83.75%	83.33%	84.38%	0.86	0.8548	89.16%	88%	90.9%	0.87	0.9303
Average	82.25%	86.67%	75.63%	**0.8548**	0.8550	**85.06%**	**88%**	**80.69%**	0.81	**0.87248**

ACC: accuracy; Se: sensitivity; Sp: specificity; F1: F1-score; AUC: area under the curve. The values shown in boldface in the table represent the maximum values for the indices.

It is seen from the table that multi-Swin outperforms the Swin transformer model for most of the indices. In the present study, we expect that this model would have greater sensitivity to detect more hypertensive patients; accordingly, the sensitivity of multi-Swin can reach 88%, which is in line with the research goals.

**FIGURE 3:**
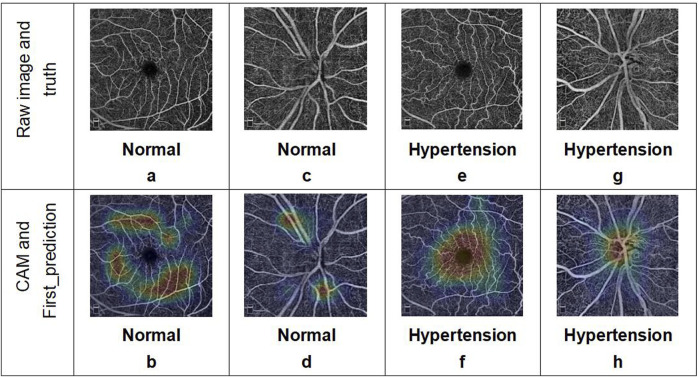
Examples of OCTA images correctly predicted by the deep-learning network. The two images in each column represent the OCTA data corresponding to a single subject: **(a, b)** macular and **(c, d)** optic disc images from a healthy volunteer; **(e, f)** macular and **(g, h)** optic disc images from a patient with hypertension. The results include the predicted categories, probability values, and corresponding class activation mapping.

At the same time, we also visualized the weights of the last self-attention blocks in the two branches of the multi-Swin network trained on the OCTA dataset through the Grad class activation mapping (CAM) method, whose results are shown in Figure 3. As seen from this figure, the model mainly focuses on the FAZ area and its surroundings as well as the ONH, while the attention points in the OCTA images of healthy people are irregularly distributed.

## 4 Discussion

In this study, a deep-learning network suitable for the research problem was designed and combined with OCTA images to distinguish hypertensive patients from healthy people based on the morphological characteristics of the fundus vessels. To the best of our knowledge, this is a pilot effort on combining deep learning with the structural characteristics of fundus vessels (especially microvessels) observed via OCTA to distinguish patients with hypertension from normal subjects. The classification results based on CNN had an average accuracy of 76.05% and average AUC value of 0.8249. In contrast, the Swin transformer showed better ability to predict hypertension based on macular OCTA images with an average accuracy of 82.25% and average AUC value of 0.855. Meanwhile, the multimodel fusion approach showed the best results, with an average accuracy of 85.06% and AUC value of 0.8719. In addition, with the Swin transformer network, the single-model (macular) branch showed better ability to predict hypertension than the single-model (optic disk) arm. Based on the evaluation indices, there is a high correlation between OCTA images and hypertension predicted using deep learning, where the sensitivity of the multimodel approach is 88%. As a significant evaluation index for preliminary screening of a disease, the sensitivity value suggests that OCTA image features recognized through deep learning can help identify changes in retinal vessels caused by hypertension more accurately and efficiently. However, direct observations of microvascular changes often cannot achieve such results. Hence, the proposed model has clinical significance for early detection and treatment of hypertension while also improving patient medical compliance. Furthermore, the drawbacks of evaluating a small number of samples can be reduced through cross-validation and data augmentation techniques. The proposed transfer learning and evaluation strategies are thus useful for analyzing small medical samples in the future.

The OCTA image results predicted by the deep-learning model were analyzed using the interpretability method. The model basis for classifying hypertension shows a certain regularity in the image features, which can explain the problems observed with the evaluation indices. The CAM visualization analysis indicates that the heatmaps in the images judged as hypertension are focused on the FAZ area and ONH, where these focus areas are relatively irregular for images judged as normal. First, this indicates that there may be individual differences in the fundus vessels of normal people that cannot be regularly identified by CAM visualization analysis in the absence of significant local lesion characteristics. Second, most of the vessels around the fovea are the tail ends of the fundus vessels and are relatively small; additionally, the thermal map of hypertension focused on this area also confirms the pathological mechanisms of hypertension noted earlier. Considering that the single model prediction of hypertension for the macular area is better than that for the optic disk, we consider that the features of the FAZ area play more important roles in the prediction. Simultaneously, we note from previous articles (Hua et al., 2020; Lee et al., 2019; Donati et al., 2021) that compared with normal subjects, the blood flow around the fovea is decreased and non-perfusion area in the fovea is significantly increased in patients with hypertension. Shin et al. (2020) observed that the perimeter of the blood vessels and total perfusion area per unit area around the ONH are lower in patients with hypertension; these findings are consistent with the results of the CAM visualization in our study and explain the significance of the image features of the optic disc in the multimodel method. Thus, we have verified the rationality and correctness of our research method, determined the direction for quantitative analysis of the OCTA image features, and provided a feature analysis method independent of statistical features for future disease diagnosis research.

Compared with a previous method of extracting and measuring specific vascular morphological features (such as curvature, fractal dimension, branching angle, and vascular aspect ratio) from fundus photography (Cheung et al., 2012), we identified the overall morphological characteristics of and changes in the fundus vessels from a higher dimension through OCTA images and deep learning, especially since the relatively small vessels cannot be observed accurately by fundus photography. The problem of parameter accuracy caused by manual identification and measurement is also solved. Furthermore, we can determine the changes in the blood vessels caused by hypertension and suggest their effects on other important organs of the body owing to the advantages of OCTA, which allows early intervention to reduce damage to other organs in the body as well as reduce the risk of prognosis. The proposed approach can also improve the efficiency and accuracy of clinical judgment of fundus vascular changes caused by hypertension when combined with deep learning.

Aside from the encouraging results, our experiments have some limitations; accordingly, follow-up research will need to consider changes in vascular morphologies and functions in the OCTA images of patients with different grades of hypertensive retinopathy (Keith–Wagener–Barker grade) (Aissopou et al., 2015) as well as OCTA changes in the blood vessels of hypertensive patients with no significant vascular changes in fundus photography. Owing to the limit on the amount of data that can be collected from healthy subjects, our dataset shows a clear imbalance with significantly more images from hypertensive individuals than healthy controls; this imbalance appears to influence the model performance as the sensitivity scores are consistently higher than specificity scores. Furthermore, the use of data from both eyes of the patients is debatable since any induced bias may be enhanced by this practice. These conditions will be improved and perfected in subsequent studies with further expansion of the sample size.

The current approach of classifying the results from pictures via the interpretable method will be used as a guide to conduct a specific analysis of the OCTA image features in our future research; to this end, we anticipate that a general explanation can be acquired from interpretation of the results of the deep-learning method to guide the OCTA image features needed to characterize hypertension.

## 5 Conclusion

Hypertensive effects are reflected in OCTA images, and the features related to hypertension can be extracted from these images by deep learning. Using the interpretable deep-learning algorithm, we analyzed the classification results of the model to explain the influences of important features from the FAZ area and ONH on hypertension. These efforts provide not only a guide for quantitative analysis of OCTA characteristics in the future but also a theoretical foundation for clinical observation of hypertension through vascular changes, allowing early intervention and treatment of hypertension as well as prediction and reduction of its systemic risks through OCTA.

## Data Availability

The original contributions presented in the study are included in the article/supplementary material; further inquiries can be directed to the corresponding authors.

## Abbreviations

OCTA, optical coherence tomography angiography; CNN, convolutional neural network; Se, sensitivity; Sp, specificity; cv, cross-validation; Acc, accuracy; ROC, receiver operator characteristic; AUC, area under the curve; HR, hazard ratio; VD, vessel density; FAZ, foveal avascular zone; ONH, optic nerve head; RNFL, retinal nerve fiber layer; CNV, choroidal neovascularization; Pr, precision; F1, F1-score; TP, true positive; TN, true negative; FP, false positive; FN, false negative; CAM, class activation mapping.
